# Locating disease genes using Bayesian variable selection with the Haseman-Elston method

**DOI:** 10.1186/1471-2156-4-S1-S69

**Published:** 2003-12-31

**Authors:** Cheongeun Oh, Kenny Q Ye, Qimei He, Nancy R Mendell

**Affiliations:** 1 Department of Epidemiology and Public Health, Yale University School of Medicine, New Haven, CT, USA; 2 Department of Applied Mathematics and Statistics, State University of New York at Stony Brook, Stony Brook, New York, USA; 3 Department of Preventive Medicine, State University of New York at Stony Brook, Stony Brook, New York, USA

## Abstract

**Background:**

We applied stochastic search variable selection (SSVS), a Bayesian model selection method, to the simulated data of Genetic Analysis Workshop 13. We used SSVS with the *revisited *Haseman-Elston method to find the markers linked to the loci determining change in cholesterol over time. To study gene-gene interaction (epistasis) and gene-environment interaction, we adopted prior structures, which incorporate the relationship among the predictors. This allows SSVS to search in the model space more efficiently and avoid the less likely models.

**Results:**

In applying SSVS, instead of looking at the posterior distribution of each of the candidate models, which is sensitive to the setting of the prior, we ranked the candidate variables (markers) according to their marginal posterior probability, which was shown to be more robust to the prior. Compared with traditional methods that consider one marker at a time, our method considers all markers simultaneously and obtains more favorable results.

**Conclusions:**

We showed that SSVS is a powerful method for identifying linked markers using the Haseman-Elston method, even for weak effects. SSVS is very effective because it does a smart search over the entire model space.

## Background

In this work, we analyzed the slope of the cholesterol increase with age in the simulated data (Problem 2). Our objective was to identify the markers that are linked to the disease genes related to a high rate of increase in cholesterol. Genetic Analysis Workshop 13 provided information that the disease genes are located on chromosomes 7(s7), 15(s8), and 21(s9), respectively, and that the gene on chromosome 21(s9) only affects cholesterol rate in the females, i.e., it interacts with gender. The Haseman-Elston [1] method allowed one to apply linear regression methods for linkage analysis. For each sibling pair, it used the number of alleles identical by descent (IBD) at each marker as the explanatory variables and a statistic measuring similarity of values of the quantitative traits in the sibling pair as the response variable. The original Haseman-Elston method [1] used the squared difference between the traits of the siblings. In a recent publication, Elston et al. [1] proposed the cross-product of the two trait values in a sib pair as the response, which was used in this paper. Suh et al. [2] applied Stochastic Search Variable Selection (SSVS), a Bayesian variable selection method proposed by George and McCulloch [3] for the linear regression model, to the Haseman-Elston method. Although the scope of Suh et al was very preliminary, with only the IBD values at the linked markers plus 10 unlinked markers used as candidate explanatory variables in the variable selection, it showed the Bayesian variable selection approach to be very promising. The study presented here extended these methods in two respects. First, we took advantage of SSVS by including all 399 markers as candidate explanatory variables. It is computationally impossible to consider all subsets of 399 markers using a traditional frequentist approach. Secondly, a hierarchical prior probability structure as discussed by Chipman [4] was imposed on the model space to study the interaction effects (epistasis). The results were reported and compared with those obtained with the more traditional forward and backward step-wise regression.

## Methods

### Haseman-Elston method

We chose to analyze the rate of change in cholesterol over time in the simulated data. First, for each individual, we obtained the least square (LS) estimate for the slope of cholesterol over the time. For the *i*^th ^sibling pair, using the LS estimate of slope as the trait (*Y*_1*i*_, *Y*_2*i*_), we computed their cross-product *CP*_*i *_= (*Y*_1*i *_-*m*)(*Y*_2*i *_- *m*) as our response values, where *m *is the mean of the slopes over all siblings in the same family. Elston et al. [1] introduced the cross-product *CP*, as the replacement of the squared difference . In our regression analysis, we adopted *CP *as the response, and also used squared-difference for comparison. For simplicity, we assumed the errors to be independent but a correlation structure could be implemented into our method in a straightforward way.

There are about 1500 full sib pairs and a few half sib pairs in each replicate. In the replicate we considered there are 1522 full sib pairs. The number of alleles shared in each pair was obtained for each sib pair at each marker using the SIBPAL program of the SAGE software [5]. There were a total of 399 markers. We had



where the ε ~ N(0, σ^2^) were assumed to be independent and *X *values were IBD scores.

To study the effect of gender, we also included the genders of the siblings as an explanatory variable. It was in fact coded as two dummy explanatory variables as follows: (male, male) = (0, 0), (male, female) = (0,1), and (female, female) = (1,1).

### SSVS

George and McCulloch [3] proposed a Bayesian model selection method for variable selection based on the Gibbs sampler. The criterion of interest was taken to be the posterior probability of a model conditional on the data that could be obtained using the stochastic search variable-selection. For the simplest case of linear regression with normal errors:

*Y *= *X*' β + ε, ε ~ N(0, σ^2^*I*),

where β may contain main effects or interactions effects. They set the prior distribution of β as mixtures of two normal distributions by introducing the latent variable γ:

β_*k*_|γ_*k *_~ (1 - γ_*k*_) *N *(0, τ^2^) + γ_*k *_*N *(0, *c*^2 ^τ^2^),

where much larger variance (*c *> 1) allowed for γ_*k *_= 1 to have a large influence. A recommended choice for these parameter values is given by George and McCulloch [3]. The value of *c *was set equal to 10 in our analysis. A model was represented by a vector γ = (γ_1_, γ_2_,..., γ_*p*_), where γ_*k *_= 0 or 1. If γ_*k *_= 0, then the marker *X*_*j *_was considered to be excluded from the model and if γ_*k *_= 1, it was considered to be included in the model. Note that β_0 _was taken to be always included, thus we could set β_0 _*N*((0, *c*^2^τ^2^). With appropriate prior on γ = (γ_1_, γ_2_,..., γ_*p*_) and σ^2^, we obtained a posterior distribution of γ using Gibbs sampling. Therefore, by examining the posterior probability of γ, we identified the optimal model with the largest posterior probability and rank the markers using the marginal distribution of each γ_*k*_. A prior for γ corresponds to a prior on the model. The commonly used independence prior implies that the importance of any variable is independent of any other variable. In other words, under this prior, each *X*_*i *_enters the model independently of the other coefficients, with probability *p*(γ_*i *_= 1) = 1 - *p*(γ_*i *_= 0) = *p*_*i*_. A smaller *p*_*i *_can be used to downweight *X*_*i *_values that are costly or of less interest. For our case, a useful reduction was to set *p*_*i *_= *p*, in which *p *is the a priori expected proportion of *X*_*i *_values in the model. When only main effects but no interaction were considered, the importance of any variable was independent of the importance of any other variable. Thus the independence prior implied that the prior of γ was simply set as prob(γ) = *p*^n^, where *n *is the number of ones in γ. Increased weight on parsimonious models could instead be obtained by setting *p *small. So in our case, *p *was set to be small, 0.02 first, and next to see how our method is robust to this choice of *p*, we chose a new value of *p *= 0.002 for comparison. The details on the MCMC algorithms can be found in George and McCulloch [3].

We applied SSVS to select markers linked to cholesterol rate from all 399 markers under consideration. Since it is impractical to track the complete posterior of γ, only the marginal posterior of each marker is obtained. Although both posterior probability of the models and marginal probabilities of each marker are sensitive to the prior settings, especially *c *and *p*, we showed that the ranking of the marginal posterior of the markers are not.

Figure 2 illustrates the robustness through plots of the ranking of the markers obtained using two different priors *p *= 0.02 and 0.002. Other prior settings showed similar high correlations in the rankings of the markers.

We followed the Markov chain Monte Carlo (MCMC) algorithms described in Chipman [4] and implemented it using the JAVA programming language. The programs were run on a Linux cluster using Intel processors. The length of the MCMC chain was set to 10,000. The running time was approximately 30 minutes on a single 1.0 GHz CPU under the above specified environment. The first 1000 samples were used as the burn-in period and not included in estimating the posterior.

### Hierarchical prior structure

When interaction effects (epistasis) are considered in the model selection, the model space becomes enormous and the common independence prior for γ is not appropriate anymore. With interactions, the prior for gamma can capture the dependence relation between the importance of a higher order term and those lower order terms from which it was formed. Chipman [4] proposed a hierarchical prior structure for this model space. The importance of the interactions such as *X*_*i*_*X*_*j *_will depend only on whether the main effects *X*_*i *_and *X*_*j *_are included in the model. This belief can be expressed by a prior for  of the form



The probability that the term *X*_*i *_*X*_*j *_is active  may take on four different values, depending on the values of the pair .



In our analysis, we set (*p*_00_, *p*_01_, *p*_10_, *p*_11_) = (0, 0, 0, *p*). This corresponded to the prior belief that if the interaction effect between two factors exists in a model, the main effects of the two factors must be included in the same model.

Our study was conducted in two stages. At the first stage, all 399 candidate markers and gender were the candidate variables in SSVS, but interactions were not considered. At the second stage, SSVS was applied to the same sib-pair responses with the top 30 candidate variables selected from the first stage and their interactions as the candidate variables. Among the third were the gender and 29 markers. This brought the total number of candidate variables in SSVS to 465. We chose only the top 30 variables from the first stage for two reasons. First, it is reasonable to assume that only a few linked loci exist and they should be contained in the top 30. Second, this is the maximum size that the current SSVS algorithm handles comfortably in the second stage.

### Step-wise regression

In order to compare the traditional method to our method, we used a step-wise method based on Akaike information criterion (AIC) [6] to select a formula-based model, which was implemented under R, the "GNU S". The details of this method can be found in the R manual [7].

## Results

**Figure 1:**
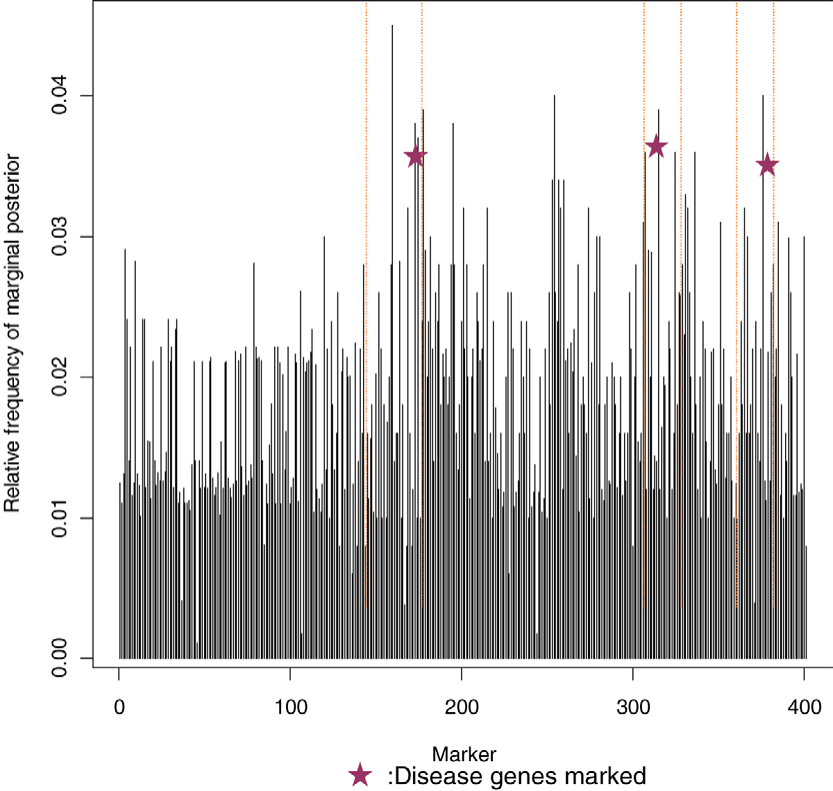
**Top ranked markers based upon SSVS **The markers are ranked according to their marginal posterior obtained using 10,000 cycles of Gibbs algorithm. Disease loci are located on chromosomes 7, 15, and 21, and gender effect is ranked at the 15^th ^(Replicate 1 of the simulated data).

**Figure 2:**
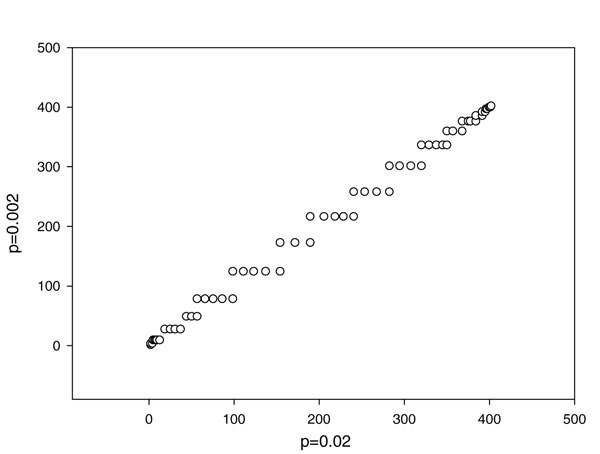
**Rankings of markers with *p *= 0.02 and *p *= 0.002 **Ranking of the markers for two different prior settings (*p *= 0.02 and *p *= 0.002) is plotted, which shows its robustness to the choice of *p*.

Only the first of the 100 simulated data sets was used. Figure 1 displays the marginal posterior of each marker obtained from SSVS with all 399 markers but no interactions. The marginal posterior was computed from the relative frequency of each markers in the MCMC sample of γ. It clearly showed that the high posterior values are concentrated on chromosomes 7, 15, and 21. Table 1 shows the top 30 markers, a marker from chromosome 7 is rated as most significant, and there are seven, four, and two markers from chromosomes 7, 15, and 21, respectively. The variable *gender *was ranked as 15^th^. Table 2 shows the most significant 20 markers obtained from the univariate LS regression and from the step-wise regression. These markers were very much evenly distributed in all chromosomes.

When we considered the results from the univariate regression, a marker from chromosome 13 was most significant. One each from chromosome 7 and chromosome 15 were only marginally significant; none from chromosome 21 (where a linked marker was located) are in the top 20 most significant markers. Similar results were obtained when backward and forward step-wise regression methods were used. Among the top 20, only two markers were from chromosome 15, and one each from chromosomes 7 and 21. Also, these two traditional methods failed to locate the gender effect as significant.

**Figure 3:**
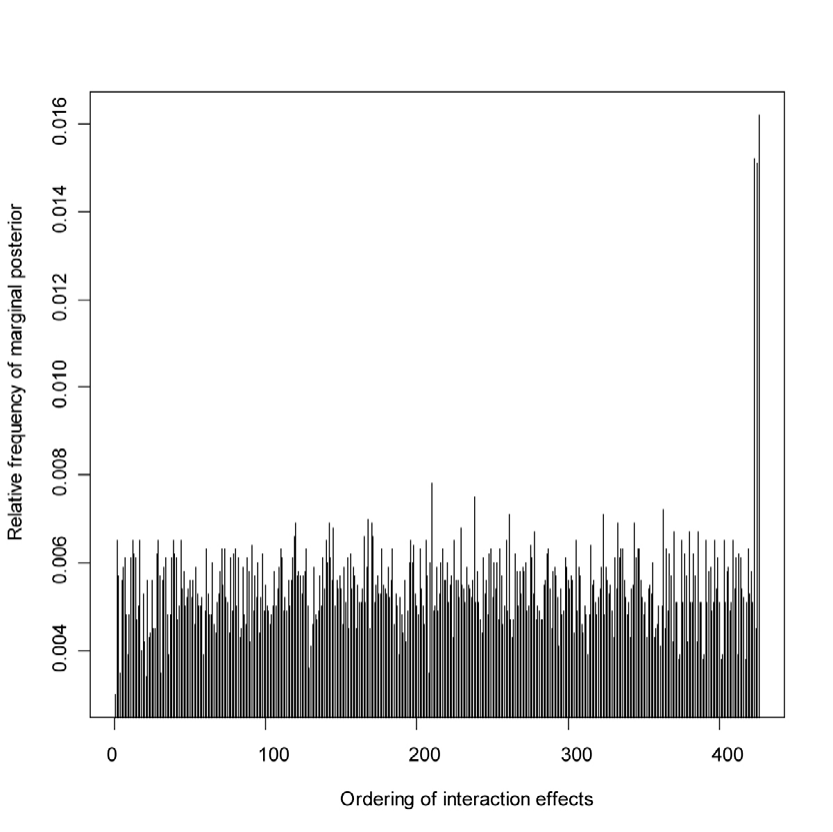
**Gene × gene and gene × gender interaction effects **The top ranked 30 markers selected from the first stage and their interactions are considered in the second stage. The interaction between chromosome 21 and gender are observed.

The result from the second stage SSVS is shown in Figure 3. The marginal posterior probabilities of the interaction effects are displayed. The existence of the gender-gene interaction on chromosome 21 is clear.

The same analysis was also carried out on the third simulated data set and similar results were obtained.

## Conclusion

We showed that SSVS is a powerful method in identifying linked markers using the Haseman-Elston method, even for weak effects. SSVS is very effective because it does a smart search over the entire model space, while the frequentist best subset model selection procedures are constrained by computing power required to examine all candidate models. The former can work on problems with many more candidate variables, which is essential when interaction effects are studied. By using the prior structures that reflect the relation among the candidate variables, SSVS can accommodate a good number of candidate markers as well as their interactions. The two-stage strategy used in this study worked well. It identified the chromosomes of the linked markers in the first stage and the interaction effects were located in the second stage. Both univariate regression and the step-wise regression failed to identify the chromosomes of the linked markers.

## Discussion

**Figure 4:**
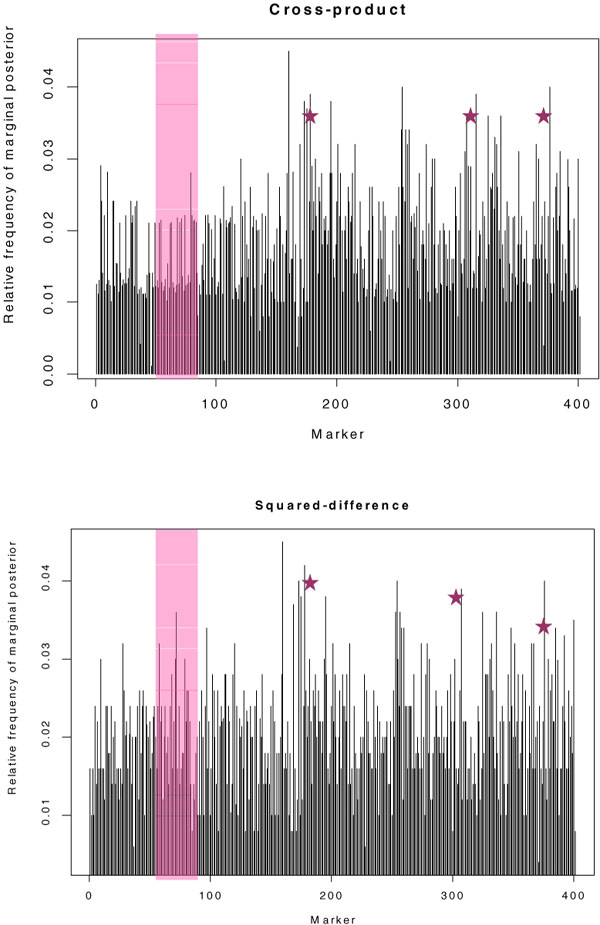
**Type I error of Haseman-Elston methods with D^2 ^and CP as responses **False positives when squared-difference is used do not overlap with ones of cross-product. This suggests that complementary information are contained in each responses.

One thing found to be interesting was that when we also use the squared-difference as response for comparison, its false positives did not overlap with those of cross-product. As we can see in Figure 4, the red strip covers those unlinked markers with high posterior probability when squared-difference is used, the posterior of these markers when cross-product are used are just average. So the information obtained from these two responses is complementary. This result is not a surprise because many recent works [8] have proposed the use of both the squared-sum and squared-difference as responses and combined the results of two regressions together in drawing the inference. As a special case, the cross-product response weights these two equally. For a comprehensive review on these "new" Haseman-Elston methods, see Feingold [8]. A natural extension of the methods proposed in this paper is to combine the posterior probability from regressions on both squared-sum and squared-difference. We will investigate this in our future research.

## Figures and Tables

**Table 1:** Top rank markers based on their marginal posterior probability.

**Posterior Ranking**	**Markers**
1	**M160 (chr7) **^A^
2	M254
3	**M315(chr15)**
4	**M173(chr7)**
tied at 5	M336, M325, **M387(chr21)**
tied at 8	M260, M257, M201, **M169(chr7)**
tied at 12	M385, M351, **M306(chr15)**
tied at 15	**400(gender)**, M367, M281, M279, M182
tied at 19	**M175(chr7)**, M120
tied at 22	**M391(chr21), M178(chr7)**
tied at 24	**M309(chr15), M179(chr7)**
tied at 26	**M311(chr15)**
tied at 27	**M164(chr7)**

^A^Disease loci are located all on chromosomes 7, 15, and 21. Markers on these three chromosomes are shown in bold face. *p *= 0.02 is used in SSVS.

**Table 2:** Top 20 markers selected in step-wise regression and univariate regression.

**Chromosome**	**Step-wise**	**Univariate**
1	M13^A^	M3
2	M57^A^	M43
3		M64^A^, M69^A^, M70^A^, M81^A^
4		
5	M114^A^	M114^A^, M128^A^
6	M142^A^	M142, M158^A^
7	M162^A^	M169^A^
8	M182^A^, M193^A^	M182^A^
9	M211^A^, M216^B^	M211^A^, M216^B^
10	M227^A^	
11	M244^A^, M253^A^	M257^A^
12		
13	M278^C^	M278^B^
14		
15	M313^A^, M318^A^	M318^A^
16		M341
17	M347^A^	M347^B^
18	M359^A^	M355^A^
19	M372^C^	
20		
21	M389^B^	
22	M397^A^	

^A^*p *< 0.05, ^B^*p *< 0.01, ^C^*p *< 0.001
